# The Treatment Experiences of Vegetarians and Vegans with an Eating Disorder: A Qualitative Study

**DOI:** 10.3390/nu17020345

**Published:** 2025-01-18

**Authors:** Courtney P. McLean, Kathleen de Boer, Megan F. Lee, Siân A. McLean

**Affiliations:** 1 Department of Psychology, Counselling, and Therapy, La Trobe University, Melbourne, VIC 3083, Australia; k.deboer@latrobe.edu.au (K.d.B.); s.mclean@latrobe.edu.au (S.A.M.); 2 Faculty of Society and Design, Bond University, Gold Coast, QLD 4229, Australia; melee@bond.edu.au

**Keywords:** eating disorders, vegetarianism, veganism, treatment, experiences, qualitative

## Abstract

Background: Vegetarianism and veganism have long been tied to disordered eating and are frequently considered to be methods of limiting available food choices. Health professionals specializing in eating disorder treatment may modify their treatment practices to support their vegetarian or vegan clients. However, there are no formally recognized clinical guidelines for the treatment of eating disorders in these groups. Moreover, no studies have yet explored the experiences of seeking and receiving eating disorder treatment while adhering to vegetarianism or veganism, which are needed to inform the development of guidelines. The present study aims to explore the lived experiences of vegetarians and vegans on eating disorder treatment through semi-structured interviews and reflexive thematic analysis. Methods: Seventeen participants (aged 19–48, 76% female, 41% vegan) with a history of receiving eating disorder treatment were recruited. Results: We identified five themes that participants described as important experiences for the treatment of their eating disorder when sought as a vegetarian or vegan: (1) Health professional perspectives, (2) The interaction of dietary status with treatment quality, (3) The give and take of treatment, (4) Lack of flexibility in treatment services, and (5) Current treatment approaches not well equipped to support dietary variations. Conclusions: This paper identifies the complex relationship between eating disorders, veganism, and vegetarianism and the perceptions of treatment from the perspectives of those who have received treatment. Our findings suggest that acknowledgement and the flexibility to work with an individual’s vegan and vegetarian values within treatment may contribute to enhanced outcomes and treatment experiences. Limitations include potential participation and response biases and a predominantly female-identifying sample. This study will contribute to the development of clinical guidelines when working with vegan and vegetarian clients.

## 1. The Treatment Experiences of Vegetarians and Vegans with an Eating Disorder: A Qualitative Study

The popularity of vegetarianism and veganism is becoming widespread and is no longer considered to be a marginalized subculture [1]. Recent estimates in Australia suggest approximately 12% of the population are vegetarian and 2% are vegan [2]. Similar proportions have also been reported in other Western countries, including the United Kingdom, the United States, Germany, and Canada [3,4,5,6,7]. Vegetarianism, described as the exclusion of meat from one’s diet, is often perceived primarily as a way of eating, whereas veganism, defined as the exclusion of all forms of animal products from one’s diet, is often considered not just a way of eating but an expression of a political philosophy or lifestyle [8]. Reasons for adhering to vegetarianism and veganism are diverse but primarily noted to be driven by animal welfare concerns, environmental sustainability concerns, and the positive health benefits of consuming more fruit and vegetables [9,10,11,12].

Vegetarianism and veganism have long been tied to eating disorders and are often considered methods of limiting available food choices or used to justify options that are lower in calories [13,14]. Indeed, vegetarians and vegans require a high degree of restraint to regulate their food intake to ensure meat and animal products are not consumed, but for many, this may not necessarily be driven by a disordered eating mindset (e.g., cognitive restraint, defined as efforts to regulate food intake to control body weight and shape) [13,15,16]. Within the literature, a recent systematic review was unable to find consensus on whether vegetarianism and veganism were associated with higher rates of disordered eating, finding similarly high rates of disordered eating across all dietary adherences (i.e., semi-vegetarian, meat-reducer, omnivore, vegetarian, vegan) [13].

Despite the overlap in food restriction, research suggests that the number of food groups excluded may not necessarily convey or contribute to the risk of eating disorder development in vegetarians and vegans, but rather, attitudes and beliefs associated with restrictive eating may be more important [13]. For instance, individuals may exclude dairy from their diet due to the sometimes inaccurate perception that exclusion will restrict calorie intake to control weight and shape, or they may exclude dairy due to environmental and animal justice reasons. Despite similar behavioral presentations, the varying underlying motivations would likely affect the extent to which the behavior constitutes symptoms of an eating disorder and, therefore, will have different treatment implications [17,18]. However, it remains difficult to examine this relationship within research and treatment settings due to purported inaccuracies across eating disorder tools in discriminating dietary restriction versus cognitive restraint [19,20,21]. The newly published Vegetarian Vegan Eating Disorder Screener (V-EDS), a novel eating disorder screening tool for vegetarians and vegans, may overcome this [15,22]. Furthermore, the use of the V-EDS may also provide an opportunity to better understand currently unknown eating disorder prevalence rates within vegetarian and vegan communities in future work.

There are currently no known formally recognized clinical guidelines for the treatment of eating disorders in vegetarians and vegans. However, anecdotal agreement within the field on approaches for eating disorder treatment includes attempting to assist the client in reducing eating restrictions of any kind [23] and often advising clients to forego vegetarianism and veganism [14]. Recent research examining the attitudes of health professionals working with individuals with eating disorders reported heterogeneous perspectives on vegetarianism, with some perceiving it as a form of restriction and others willing to work within a client’s dietary adherence [24]. Health professionals further acknowledged that for some clients, vegetarianism may form part of their identity, and this can be especially challenging when working within a treatment setting that promotes the reintroduction of all food groups. Such research suggests that treating individuals with eating disorders and providing scope to adhere to vegetarianism or veganism can be challenging from a health professional perspective, yet limited research has examined the perspective of vegetarian and vegan eating disorder treatment seekers [24].

Further research is needed to understand the impacts of adhering to vegetarianism and veganism on treatment experiences from a lived-experience perspective. Understanding such perspectives can provide valuable clinical opportunities, including informing whether current eating disorder treatment practices are meeting the needs of vegetarians and vegans seeking support, what improvements could be made, and the approaches of treating health professionals. Such findings may also be instrumental in the development of formal clinical guidelines for these groups in the future. Therefore, the present study aimed to explore the lived experiences of vegetarians and vegans on eating disorder treatment.

## 2. Method

### 2.1. Study Design

A qualitative phenomenological research design was used to capture participants’ perspectives of receiving eating disorder treatment as a vegetarian or vegan, using semi-structured interview discussions. The reporting of this study adhered to the guidelines outlined in the COnsolidated criteria for REporting Qualitative research (COREQ; [25]). See the Supplementary Materials for the COREQ checklist. This study obtained approval from La Trobe University Human Research Ethics Committee (HEC24087, 30 April 2024).

### 2.2. Methodology

A phenomenological approach is most suited to this research design to understand the lived experiences of participants, as it encourages the exploration of complex and personal phenomena such as eating disorder treatment and adherence to diet types tied to identity [26,27]. The phenomenological perspective acknowledges that vegetarians’ and vegans’ experiences are shaped by their interactions with society, social support systems, and personal morals and beliefs. Second, phenomenology ensures that the phenomenon can be explored through the participants’ perspectives whilst reducing researcher bias through reflexivity [26].

An integral aspect of reflexive thematic analysis involves considering the author’s positioning throughout the data analysis process [28]. The research team included the first author, an early-career researcher in eating disorder and vegetarian/vegan research. The second author is an early-career researcher and clinical psychologist in eating disorders, and the third author is a researcher in nutritional psychiatry in vegetarian/vegan mental health, while the fourth author is a senior researcher in the eating disorder field. Personal dietary adherence across authors ranged from vegan, flexitarian, pescatarian, to omnivorous. Thus, all authors contributed unique and important perspectives to the research. Data coding was evenly distributed between the first and second authors. To address potential researcher bias, self-reflexive methods were integrated into the analysis processes, including maintaining reflexive journals and regular discussion of coding processes.

### 2.3. Participants

Participants were recruited using purposive and targeted sampling techniques, primarily through national and state-wide eating disorder organizations and professional networks of the research team. The inclusion criteria required participants to be 18 years or over, living in Australia, and have previously had eating disorder treatment whilst adhering to a vegetarian or vegan diet. Treatment was defined as any formal treatment sessions for psychological treatment or dietetic health services previously received. Participants must have undertaken at least one treatment session to be eligible for the study. There were no restrictions on the treatment period. There was no relationship with any of the participants prior to the study’s commencement.

A total of 17 participants took part in the present study. The sample size was sufficient to allow for in-depth exploration into a novel area and generated rich data across several characteristics including dietary adherence, eating disorder diagnosis, and treatment type [29,30]. As such, data saturation was reached for the present study. The average age was 27.25 years (*SD* = 6.94; range = 19–48); most participants identified as female (76.47%) and had varying eating disorder diagnoses at the time of seeking treatment as a vegetarian or vegan. See Table 1 for additional participant characteristics.

### 2.4. Procedure

Individuals were directed to an expression of interest survey to learn more about the study and complete a screening survey held on REDCap. First, the participants read the explanatory statement and provided informed consent. The screening survey included questions about demographic characteristics, eating disorder diagnosis when treatment was sought as a vegetarian or vegan, length of diagnosis, type of health professional/s where psychological and/or dietetic treatment was received, whether in-patient care services were received, and vegetarian/vegan status at the time of seeking treatment. Individuals who met the inclusion criteria were invited to participate in the study via email and were provided with a copy of the explanatory statement and consent form and the interview guide upon invitation.

Participants met with CM to take part in the interviews, which were conducted online using the secure video conferencing software Zoom. To begin, participants were advised of the purpose and aim of the research and the elements of consent were verbally reviewed with the participants prior to the start of the interview. A brief interview guide was flexibly used throughout the interviews, integrating both open-ended questioning and probing techniques, including “*How was your [vegetarianism, veganism] perceived by your health professional/s*”. See Supplementary Materials for the interview guide. All interviews were conducted in English and lasted an average of 31.28 min (*SD* = 7.68). The interviews were conducted between June and August 2024. No repeat interviews were conducted.

### 2.5. Analysis

All interview recordings were transcribed using Otter.ai, reviewed for accuracy of transcription and anonymized by the research team (CM and KDB). The participants were emailed their transcript and asked to amend any inaccuracies and return their transcript within two weeks. The final transcripts were independently coded by two researchers (CM and KDB) using NVivo and employing reflective thematic analysis guided by Braun and Clarke [31,32]. Initially, the coders conducted two practice interviews, whereby they independently coded the interviews, and then met to assess agreement. Once the coders agreed with their processes, the remaining interviews were divided randomly and equally between CM and KDB for analyses. The first stage of reflective thematic analysis involved deep *familiarization* with the data through reading the transcripts. In the second stage, *initial coding*, an open coding approach to label segments of text within the data set was used. In stage three, *generating themes*, codes were grouped into categories according to a central organizing concept to generate the main themes through discussion between CM and KDB. In stage four, *reviewing themes*, to ensure a rich understanding of the data, researchers SM and ML independently reviewed the drafted themes and provided feedback and impressions on possible meanings, leading to stage five, *theme finalization*. Finally in stage six, *interpreting and reporting*, the researchers continued to revise and review code labels, codes, and themes during the write-up phase.

## 3. Results

We identified five superordinate themes that participants described as important experiences in the treatment of their eating disorder when received as a vegetarian or vegan, all with subthemes. See Supplementary Figure S1 for a flowchart of the themes, subthemes, and their definitions. Prior to presenting the themes, we acknowledge that many participants recognized the linkage between eating disorders and vegetarianism and veganism.

*“* *I know that for people with an eating disorder, or with a history of an eating disorder…, placing dietary restrictions can trigger people and could be detrimental.” (P1, Vegan)*

*“I would love to go vegan again in more of a sustainable way, but I can recognize now that with my eating disorder that it wouldn’t be sustainable. And I feel my eating disorder would latch on to it and use it as a way to restrict.” (P8, Vegetarian)*

Given the egosyntonic nature of eating disorders and the values-based motivation for veganism and vegetarianism, such insights are important considerations in the context of the results.

### 3.1. Theme 1. Health Professional Perspectives

This theme captures the ways in which treating health professionals’ perspectives on vegetarianism or veganism were perceived to influence treatment for the participant. Two sub-themes were identified: (i) Attitudes of health professionals towards dietary status, and (ii) Knowledge and skill of health professionals.

#### 3.1.1. Attitudes of Health Professionals Towards Dietary Status

Health professionals’ attitudes towards their client’s vegetarian and vegan dietary status varied. Some participants noted they saw health professionals who refused to work with them due to their dietary adherence.

*“But on the whole, all of the dietitians that I came across were not open to it at all… Basically, anything that wasn’t a diagnosed allergy, you had to eat it regardless. And the title of vegan or gluten free or anything like that was pretty much dismissed straight off the bat.” (P11, Vegan)*

This was often coupled with a perceived lack of trust in vegetarians and vegans seeking treatment, whereby health professionals were thought to be suspicious or distrusting of their clients. One participant (P13, Vegetarian) described:

*“[Vegetarianism] wasn’t really taken seriously… They thought that I was tricking them, kind of trying to be sneaky, and see how I could consume the right amount of food in the smallest quantity.”*

Alternatively, some participants reported that the health professionals they worked with were open to working within their veganism and vegetarianism:

*“My [health professionals] have all been pretty respectful of my choice to be vegetarian… My current dietitian is definitely very, very understanding.” (P8, Vegetarian)*

#### 3.1.2. Knowledge and Skill of Health Professionals

Similarly, the perceived degree of knowledge and skill regarding vegetarianism and/or veganism in eating disorders held by health professionals varied widely across participants. Some noted the health professionals they saw had the necessary expertise specific to vegetarian and vegan diets within an eating disorder context, while others did not.

*“I feel like my current dietitian is definitely very knowledgeable about vegetarianism and veganism and eating disorders.” (P8, Vegetarian)*

*“Overall, the standard of knowledge was not really there… Throughout the years that I’ve gone in and out of treatment, there’s the understanding or perception that you can’t recover fully if you’re vegetarian or vegan.” (P6, Vegetarian)*

*“I feel like [my] psychiatrist has some really warped views on health and healthy eating and things like that.” (P16, Vegetarian)*

Many participants called for more education and professional development opportunities for health professionals to improve their expertise in the field. One participant (P13, Vegetarian) commented:

*“If they’re going to take veganism seriously as a lifestyle choice moving forward, they need to be better equipped with dietitians and nutritional information to provide adequate service to those patients…”*

A perceived lack of expertise specific to vegetarianism and veganism during treatment meant that many participants found recovery difficult once treatment ceased. Participants noted they missed out on tailored education around how to be safely vegetarian or vegan in recovery, leaving them unprepared to continue recovery outside of treatment.

*“I really wanted to have information on how I could stay being vegetarian whilst getting the most nutrients. And it could have just been the dietitian that I saw, but it wasn’t really taken seriously… if I’m going to try and get better, I need to know how to eat properly as a vegetarian.” (P13, Vegetarian)*

*“When I did eventually actively start trying to go into that recovery phase, I didn’t have any point of reference, because not a single person that I’d been working with had attempted to work with me within the constraints of veganism, and so it was really tough, because all of the meal plans and the diet lists and stuff that they give you, not a single one of them is tailored towards veganism.” (P11, Vegan)*

In sum, this theme describes the varying degrees of attitudes, knowledge, and skill of health professionals towards vegetarianism and veganism. Limited knowledge and skill specific to working with these groups may be driven by limited educational opportunities for health professionals or a lack of consensus within the field as to how these dietary choices interact with eating disorders. This perceived knowledge and practice gap appeared to be transferred to clients in terms of a lack of tailored vegetarian and vegan-related psychoeducation for eating disorders.

### 3.2. Theme 2. The Interaction of Dietary Status with Treatment Quality

This theme describes the impact of the participants’ dietary adherence on the quality of care of their treatment. Participants emphasized they perceived their quality of care to be lower due to adhering to a vegetarian or vegan diet. This was frequently noted to be caused by conflict with their health professionals, and subsequent setbacks in building a therapeutic relationship.

*“I do think that it probably would have been better quality of care if I wasn’t a vegetarian. Because I just would have had less conflict.” (P16, Vegetarian)*

With relatively similar frequency, others noted no difference in the impact of their quality of care due to adhering to a vegetarian or vegan diet. One participant (P8, Vegetarian) noted the following:

*“But I feel like my vegetarianism didn’t really make a difference to the quality of my care, other than getting a little bit of hesitation from professionals about being vegetarian.”*

#### The Role of Additional Support in Improving Quality of Care

Participants emphasized the impact of family and loved ones who advocated for their dietary adherence within treatment, and in turn, supported greater quality of care.

*“[My parents] did advocate for me because they could see that it was important because I’d already been vegetarian for three years prior to my eating disorder starting.” (P8, Vegetarian)*

*“But I think that was due to my mum’s advocacy and dedication to keeping me vegetarian and that she was in charge of my meals and food. So I wasn’t relying on a hospital system or an out-patient kind of system to provide it.” (P10, Vegetarian)*

In sum, this theme describes the impact of vegetarian and vegan adherence on subsequent treatment quality. Negative impact was most frequently attributed to conflict within the therapeutic relationship but protected by a loved one’s involvement in the treatment journey.

### 3.3. Theme 3. The Give and Take of Treatment

This theme explores the compromises that participants made regarding their dietary adherence throughout treatment. While participants acknowledged there are already challenges associated with eating disorder recovery, many identified additional difficulties specifically ascribed to the desire to maintain their dietary adherence.

#### 3.3.1. The Need for Self-Advocacy

Often participants described needing to prove or justify their dietary status to health professionals. This was achieved in many ways, such as demonstrating to their treatment team that they could achieve weight restoration, providing food logs, or other evidence of vegetarian/vegan timelines alongside their eating disorder development.

*“The first couple of admissions that I had were under the Maudsley treatment program. And they were like, ‘You can’t be a vegetarian unless you have been vegetarian for five years prior to being admitted, or had religious reasons’, and none of which applied to me.” (P2, Vegetarian)*

*“I had to be able to prove to them that eating a plant-based diet wasn’t restrictive, that you could still have all the foods that you wanted to have and show them that you could thrive on a vegan diet.” (P1, Vegan)*

Participants described this experience as needing to fight or battle for their vegetarian or vegan values. One vegan participant (P11) noted:

*“Every single admission I’d go in, and we’d have the battle over whether or not I had to eat fish, or whether or not I had to eat egg… There were a couple of admissions where I literally didn’t speak because I decided that they could force me to physically be there, but they couldn’t force me to participate.”*

#### 3.3.2. The Punitive Nature of Treatment

It was noted that participants were often made to feel by their health professional as though they were failing treatment and recovery by adhering to vegetarianism or veganism, similar to feeling as though they were doing something “wrong”.

*“As well as psychologists be like, ‘Well, if you’re not willing to start eating meat again, then you’re not serious about recovery, so why should I help you’… I had one [psychiatrist] tell me that I would never be able to recover because I was never going to get the nutrition that I needed from a vegetarian diet.” (P16, Vegetarian)*

At times, participants noted their health professional made them feel like a “difficult” patient due to their dietary status. One vegetarian participant (P3) said:

*“There was an in-patient unit that I only lasted like 3 h in… I did feel as though I was being judged or viewed as a difficult patient for wanting vegetarian options.”*

#### 3.3.3. An Ultimatum

In some settings, participants felt as though they needed to choose between their dietary status or receiving treatment, feeling as though they were faced with an ultimatum.

*“* *I think [it] would also put some people at sort of an ultimatum of either seeking treatment or being able to continue with the lifestyle that they view as being ethical.” (P1, Vegan)*

However, many participants did compromise on their dietary status to receive treatment, but this was generally only for the period of treatment, with many reverting back to a way of eating that aligned with their morals and values.

*“… With the mealtimes, I kind of just had to get through it to stay in the treatment. And I think in that way I didn’t really get to be mindful while eating or actually work through that distress because I was partially dissociating through all of that.” (P5, Vegan)*

#### 3.3.4. Negative Consequences of Compromising on Dietary Status

It was a common experience for participants to have negative physical and psychological consequences from needing to compromise on their vegetarian or vegan dietary status. Some noted that their veganism was also driven by dairy intolerance but believed their health professionals saw this behavior as a means to further restrict, requiring them to consume certain foods, such as dairy, which made them physically unwell.

*“* *It does turn out that I am lactose intolerant… And since now that I’ve removed it from my diet; it hasn’t cured things, but it has helped reduce the pain, so my quality of life has increased. And so for 3 years I was consuming dairy because I thought, ‘Well, if I cut this out, then I’ll be failing at eating disorder treatment. That will be another problem instead of a solution’.” (P10, Vegetarian)*

Others noted that the psychological consequences of needing to compromise on their dietary status, as well as their morals and ethics, made their recovery process harder. Some commented that needing to compromise on their dietary status meant they were unable to fully engage in their treatment—they felt as though they rushed through treatment to be able to go back to being vegetarian or vegan:

*“I might have stayed in treatment a little bit longer or might have progressed more genuinely if I was allowed to be vegan… I really pushed myself through all the phases [of treatment].” (P5, Vegan)*

In sum, this theme describes the concessions needed during treatment, with many describing the need to justify their dietary status to their health professional. Others criticized aspects of treatment as punitive, expressing they were made to feel like they were doing something “wrong” by adhering to vegetarianism or veganism. Often, participants were required to forgo their dietary adherence to receive treatment, which was associated with negative physical and psychological consequences.

### 3.4. Theme 4. Lack of Flexibility in Treatment Services

Overall, participants emphasized there was a general lack of flexibility within their treatment service. However, there appeared to be variations in flexibility across settings and health professional types. For example, receiving treatment through in-patient services was noted to be the least flexible in accommodating vegetarianism and veganism compared to out-patient services.

*“I’ve also done a couple of in-patient stays where I have not been allowed to be vegan. So those have been probably the hardest parts of treatment for me.” (P5, Vegan)*

#### 3.4.1. Feelings of Lack of Autonomy

This lack of flexibility in treatment services led many to feel a lack of autonomy throughout their treatment journey.

*“* *Providing autonomy and choice and giving people control within a really uncontrollable environment for them is so important, as is respecting needs. Like if someone comes in and needs halal food, do we question that? If it’s a religious choice-they’re not incapable of eating it, they just choose not to. And I don’t see why veganism should be any different.” (P11, Vegan)*

*“I think I get really stuck when someone says no-no one likes being told what to do. And especially because I’ve been on involuntary treatment, I just don’t like it. So, I always want the option to have the choice.” (P6, Vegetarian)*

#### 3.4.2. Impact of Limited Meal Flexibility in In-Patient Settings

Within in-patient settings, participants commented on the impact of having reduced or limited meal flexibility due to their dietary status. For example, many noted that meat was classified as their “dislike/s”, meaning there was no additional room for other food accommodations.

*“* *With the three dislikes, it meant that I only really had two dislikes. And I felt like it was a little bit unfair. And it meant that I had to eat some foods that I didn’t like, and then that would have caused me extra distress because I didn’t have that extra little bit of flexibility that other patients would have.” (P8, Vegetarian)*

Participants felt that the available vegetarian or vegan meals in in-patient settings were generally not as nutritionally diverse compared to their omnivore counterparts. This limited variety meant that individuals were not able to challenge some fear foods related to their eating disorder. Some participants mentioned:

*“I think there was less consideration when it came to food as a vegetarian. There was not as much thought into the meals when we had to eat meals all together, the vegetarian option was always pretty much the same. And even within that, there wasn’t [sic] as many challenges.” (P13, Vegetarian)*

*“ln every hospital, you need to meet your two proteins, vegetables, [and] dairy, and there’s no way about it… I think they need to be more aware of alternatives.” (P6, Vegetarian)*

*“… For the vegetarians, there wasn’t as much diversity in the food options, which looking back on it now they weren’t giving us much of a challenge.” (P13, Vegetarian)*

In sum, this theme describes the varying degree of flexibility in eating disorder food offerings in treatment services, which frequently led to feelings of a lack of autonomy. The impact of a lack of flexibility in treatment services was most felt within in-patient settings, with reduced or limited meal options for vegetarians and vegans.

### 3.5. Theme 5. Current Treatment Approaches Not Well Equipped to Support Dietary Variations

This theme describes the participants’ reflections on the current treatment guidelines, which were perceived to conflict with recovery for vegans and vegetarians. For some, this made treatment engagement and recovery more challenging, as not only were they asked to overcome their fears related to shape and weight, but also to compromise on parts of their identity and value structures.

#### 3.5.1. Black and White Model of Treatment

Participants noted that eating disorder treatment is often rigid, meaning that vegetarianism and veganism are generally not accommodated. Participants frequently spoke to the “cookie cutter” model of treatment as being unhelpful for the complexity of eating disorders and vegetarianism/veganism.

*“* *I feel like [health professionals] had a textbook knowledge of eating disorders, but what they lacked was the ability to recognize that every single eating disorder is different, and they lacked the capabilities of applying those differences to each person that they were presented with.” (P11, Vegan)*

*“There seems to be a bit of a textbook or cookie cutter way to do recovery. I think the issue is that it’s the same. As in they use the same approach and rules as someone who is an omnivore.” (P14, Vegan)*

#### 3.5.2. Vegetarian/Vegan Values in Treatment

Participants commented that they were surprised at being unable to lean into their vegetarianism or veganism, considering the values-based model of eating disorder treatment. One participant (P14, Vegan) explained:

*“You’re supposed to go towards your values and your identity outside the eating disorder… I felt [my veganism] would be a positive recovery choice. And then that wasn’t really allowed.”*

Some participants found other ways to align themselves with their vegetarian/vegan values, beyond solely what they eat:

*“Although I wasn’t allowed to be vegan, there were sometimes outings to, for example, [redacted]. And I didn’t go into the [animal-based entertainment centre] because that’s not in line with my values… I just tried to find different ways to still try to live by those values, even though it still felt like I was going against that a bit.” (P5, Vegan)*

In sum, this theme reflects that current treatment guidelines are rigid and do not consider the unique and specific needs of vegetarians and vegans in recovery. Many participants assumed that the values-based model of treatment may have supported vegetarian and vegan principles but found other non-food ethical principles to align themselves with during recovery (e.g., cruelty-free cosmetics, excluding wool and leather, rejection of animal-based entertainment).

## 4. Discussion

This study aimed to qualitatively explore the lived experiences of vegetarians and vegans in eating disorder treatment. Understanding these experiences can provide valuable clinical opportunities to inform whether current eating disorder treatment practices are meeting the needs of vegetarians and vegans seeking support, what improvements could be made, and the approaches utilized by treating health professionals. We identified five themes that participants described as important experiences for the treatment of their eating disorder: (1) *Health professional perspectives*, (2) *The interaction of dietary status with treatment quality*, (3) *The give and take of treatment*, (4) *Lack of flexibility in treatment services*, and (5) *Current treatment approaches not well equipped to support dietary variations.*

The role of health professionals in the interaction of eating disorders and vegetarianism and veganism was overall varied. It was clear that the attitudes of health professionals played a large role in the solace and trust of the client/practitioner relationship. Participant perspectives noted that negative health professional attitudes to vegetarianism and veganism may be perpetuated by a lack of education or skill. However, we note this may also be driven by biases or a clash of values. There is one known consensus statement written in collaboration with the Royal College of Psychiatrists, the British Dietetic Association, and the United Kingdom’s national eating disorder charity, BEAT, on the nutritional considerations for treating vegan eating disorder patients [33]. However, there are no formally recognized treatment guidelines for these groups, leaving health professionals without guidance around best practice. This perceived lack of expertise specific to vegetarianism, veganism, and eating disorders was thought to impact clients, with many participants noting they missed out on tailored education around how to be vegetarian or vegan in, or post, recovery. This was commonly experienced through recovery meal plans, whereby participants were not informed of comparable and adequate vegetarian or vegan alternatives (e.g., Is coconut, almond or soy yogurt a recommended alternative to dairy yogurt? Does 150 g of chicken nutritionally equate to 150 g of tofu?).

Some participants reported lower treatment quality due to their dietary adherence and how their health professionals perceived such adherence. This was mostly due to disagreements between client and practitioner, with some participants noting they felt like they were a “difficult client”. Often this led to conflict, impacting the therapeutic relationship. The literature demonstrates that the therapeutic relationship is a key predictor of eating disorder treatment outcomes, including achieving a target weight [34] and reducing premature dropout rates [35]. When tied with particularly high treatment dropout rates among eating disorder populations [36], this may place individuals adhering to vegetarianism and veganism as a particularly at-risk group. The literature has demonstrated that a validating, empathic, and genuinely collaborative approach may enhance the therapeutic relationship when working with clients with eating disorders [37], and this may be particularly relevant when working with vegetarian and vegan groups [14,15]. It is important to note that previous research exploring the treatment experiences in people who do not adhere to vegetarianism or veganism has also highlighted the importance of connectedness between client and practitioner [38,39]. For example, participants noted that inconsistency in the therapeutic alliance weakened their path to recovery, disrupting trust with their health professional and reducing engagement in treatment [38]. Therefore, the value of a strong therapeutic alliance may not be a vegetarian- or vegan-specific treatment experience, but perhaps these dietary variations may place additional vulnerabilities on the relationship due to tension or disagreements in shared decision-making [40].

Participants described their eating disorder treatment as a period of compromises and making mutual concessions with their healthcare team. Frequently these experiences were noted as attempts to negotiate food restrictions with their health professionals. More broadly, negotiating treatment goals may be a common treatment perception in non-vegetarian and -vegan clients [41]. However, negotiations in this study were more commonly focused on specific food consumption, akin to consuming a dairy-based Fortisip but not the consumption of any meat in people adhering to veganism. However, we do note the recent release of Fortisip PlantBased, which may provide vegans with an avenue to engage in the nutritional aspects and requirements of treatment without compromising on their veganism. As highlighted in the interviews, attempts to negotiate with a health professional often meant that clients needed to self-advocate or rely on the advocacy of family members or loved ones. However, assertive communication may not always be possible in eating disorder recovery; self-advocacy is difficult, particularly when one is unwell and communicating one’s needs does not necessarily mean they will be respected [42,43].

Negotiations with a healthcare team were not always possible for many participants; often participants had to choose between maintaining their dietary adherence or receiving treatment for their eating disorder. This is of particular concern, given the barriers associated with accessing appropriate and specialized care in the first place [44]. Additionally, within the literature, there is growing support for the assertion that eating disorders are malleable over time, whereby eating and weight behaviors may become more ingrained the longer they are left untreated [45]. Delayed intervention and longer duration of illness have been associated with poorer outcomes, which may be particularly prevalent in vegetarian and vegan groups [46,47].

Our findings call for additional flexibility within eating disorder treatment for vegetarian and vegan groups. We found that flexibility varied greatly depending on profession, treatment setting, and dietary adherence. Indeed, participants who sought treatment for their eating disorder through in-patient services noted these to be more rigid and unwilling to modify treatment practices to accommodate individual dietary adherences, relative to other treatment services such as out-patient or private practice. However, for eating disorders that involve restriction (e.g., anorexia nervosa), treatment guidelines recommend increasing food intake and variety and thus reducing restrictive behaviors [23]. Therefore, increasing the flexibility of services to allow for more restrictive ways of eating does conflict with eating disorder treatment principles. Rigidity of treatment also appeared to differ between vegetarians and vegans, akin to a spectrum that reflected the number of food groups excluded (i.e., omnivorous, semi- or flexi-vegetarian, vegetarian, vegan). While eating disorder treatment approaches more broadly may be perceived to be one-size-fits-all [41,48], this sentiment appeared to be accentuated among vegetarian and vegan groups.

Current treatment processes are currently not established to support vegetarian and vegan clients, with such rigidity in treatment meaning that many vegetarian and vegan clients feel as though they are not accommodated. Indeed, additional research is needed to explore how vegetarian or vegan values may be integrated into treatment models used to guide recovery, which may be particularly salient for in-patient services. For example, it would be useful to identify a range of vegetarian and vegan values (e.g., validating dietary adherence, adapted meal plans, maintenance of vegetarianism/veganism) that sit along a continuum from *healthy* to potentially *problematic*, and how they could be integrated into treatment processes depending on the presentation of the client. Further to this, participants noted that a lack of flexibility led to feelings of loss of autonomy. We acknowledge it may be difficult to balance an improvement in the client’s nutritional status while ensuring autonomy is provided, particularly in cases in which weight restoration is required. Guidelines and training should support health professionals to offer treatment options that maintain the client’s autonomy and facilitate open conversations around the client’s dietary adherence in an open and nonjudgemental way [13,19].

## 5. Strengths and Limitations

This study presents several strengths. To our knowledge, this is the first study to explore the experiences of vegetarians and vegans engaging in eating disorder treatment. Using a qualitative study design, the study provided foundational insights that may contribute to the development of clinical guidelines and treatment recommendations. Furthermore, diversity among the sample in terms of eating disorder diagnosis, treatment settings (out-patient, in-patient), and health professionals they worked with (psychologists, dietitians, psychiatrists) provided a rich understanding regarding how experiences may align.

Despite these strengths, it is important to note potential limitations. First, it is possible that participation bias may have skewed the findings, whereby participants who had experienced negative treatment encounters may have been more motivated to participate in the present study. Furthermore, the participants were primarily recruited through eating disorder organizations and charity networks and may be engaged members of these communities, leading to potential response biases. For example, individuals who have had more negative treatment experiences might be more likely to engage, advocate, or be a member of eating disorder organizations and charities. Indeed, future research should endeavor to recruit through more diverse sources, including through community networks or treatment facilities. Next, the present findings are based on a sample of women and non-binary individuals and are, therefore, limited in the extent to which they can be generalized to other genders. While it was valuable to have several non-binary individuals take part, a group who are typically underrepresented in eating disorder research, extending the findings to males and other gender-diverse groups is needed in future research to explore potential gender-based differences. Despite our specific and targeted recruitment efforts towards males, recruiting men with a lived experience of seeking eating disorder treatment as a vegetarian or vegan proved to be extremely difficult. Furthermore, the literature demonstrates differences in motivations for dietary adherence and attitudes to plant-based products between Western and Eastern countries [49]; therefore, it remains important to explore this area of work cross-culturally. Understanding how cultural differences may impact the prevalence of vegetarian or vegan eating disorder rates may also be of clinical interest. Finally, the findings from the present study are preliminary in nature, and future research should work to validate the generated themes through quantitative research.

## 6. Clinical Implications

This paper provides a useful stepping stone to begin to think about how to best support vegetarians and vegans along the eating disorder treatment journey. It is recommended that health professionals spend time exploring a client’s motivation for adhering to vegetarianism or veganism in a compassionate and non-judgmental manner. Given vegetarianism and veganism can be tied to an individual’s identity and values, consideration should be given as to whether challenging their dietary adherence is conducive to recovery. Similarly, gaining clarity within treatment on the extent to which the restriction of intake of certain foods relates to values and identity, as opposed to eating disorder behaviors, is important for the appropriate formulation of treatment. Health professionals working in the eating disorder space may also consider the use of the V-EDS as a screening tool in treatment to facilitate this understanding [15]. Specifically, items one to six of the V-EDS (i.e., the dietary characteristic items) provide a novel distinguishing feature that may assist health professionals in understanding a client’s dietary status and how it may impact their eating disorder symptoms. These items are developed as a “conversation starter”, offering a neutral and non-assumptive way to query dietary adherence [15]. If accompanied by an eating disorder diagnosis, these items may provide insights into whether treatment modifications may be considered to reduce additional distress. For example, Item 5 (“*You are willing to introduce meat to your diet if it is vital for your survival*”) may be useful to assess a client’s receptiveness to eating meat and whether modifications to the recovery plan should be considered [15,22].

If a client’s vegetarianism or veganism is not grounded in an eating disorder mindset, health professionals should work with the client to develop recovery plans and strategies that are in line with their dietary values. This may be especially relevant for dietitians. Indeed, there are longer-term, systematic issues that need to be addressed in the field. The development of clinical guidelines remains a critical research avenue and may be used to inform how in-patient programs could review their current approaches to ensure best practice for these groups. Furthermore, there is a clear need for professional development resources for health professionals to learn about the assessment and treatment of eating disorders in vegetarians and vegans. These should include bias-reduction strategies, knowledge in approaching vegetarianism and veganism in sensitive and non-judgmental ways in treatment, and education on plant-based nutrition.

## 7. Conclusions

In conclusion, this study provides insights into whether current eating disorder treatment practices are meeting the needs of vegetarians and vegans seeking support, along with potential improvements that could be made to support these groups. This study recruited 17 participants with a history of receiving eating disorder treatment to qualitatively describe their experiences of treatment when sought as a vegetarian or vegan. Our findings highlighted several aspects of the treatment journey that were influential to their treatment. Specifically, findings related to the acknowledgment of vegetarian/vegan perspectives in treatment and the alignment of personal values have important implications regarding the treatment of individuals within these groups. Together, the results of the present study indicate a significant gap in eating disorder research. Additional research seeking to understand the perspectives of health professionals in working with vegetarian and vegan clients with eating disorders would be of value and may provide an avenue to explore how vegetarianism and veganism could be safely and effectively incorporated into clinical eating disorder practice to better support these individuals.

## Figures and Tables

**Table 1:** Participant characteristics.

Characteristic	Sub-Category	*n* (%)
Gender	Female	13 (76.47)
	Non-binary	3 (17.65)
	Prefer not to disclose	1 (5.88)
State of residence *	New South Wales	4 (23.53)
	Victoria	8 (47.06)
	Queensland	2 (11.76)
	Western Australia	1 (5.88)
	Tasmania	2 (11.76)
Eating disorder diagnosis *	Anorexia nervosa	6 (35.29)
	Atypical anorexia nervosa	4 (23.53)
	Binge eating disorder	1 (5.88)
	Bulimia nervosa	2 (11.76)
	EDNOS	1 (5.88)
	Multiple diagnoses	3 (17.65)
Eating disorder duration	1–5 years	7 (41.18)
	6–10 years	4 (23.53)
	11–15 years	4 (23.53)
	16–20 years	0 (0.0)
	More than 20 years	2 (11.76)
Received in-patient care	Yes	8 (47.06)
	No	9 (52.94)
Vegetarian/vegan status	Vegetarian	8 (47.06)
	Semi-vegetarian	1 (5.88)
	Pescatarian	1 (5.88)
	Vegan	7 (41.18)

* Rows in sub-category may not add up to 100 due to rounding.

## Data Availability

Data are contained within the article.

## Supplementary Materials

The following supporting information can be downloaded at: www.mdpi.com/article/10.3390/nu17020345/s1. The COREQ checklist and the interview guide are available in the Supplementary Materials. A visualization of the themes, subthemes, and their definitions is available in Figure S1: Identified themes, subthemes, and their definitions.
