# Supplementary material for: The Treatment Experiences of Vegetarians and Vegans with an Eating Disorder: A Qualitative Study
Source: Nutrients. 2025 Jan 18;17(2):345. doi: 10.3390/nu17020345 (PMC11768365; doi:10.3390/nu17020345)
Supplement: Supplementary file 1 [file nutrients-17-00345-s001.zip › nutrients-3403981-supplementary/Supplementary Materials - Interview Guide.pdf]

Interview Guide

1. You were previously [vegetarian, vegan], what was/were your motivation/s to go [vegetarian, vegan]?
    - How long were you [vegetarian, vegan]?
2. And when abouts into your [vegetarianism, veganism] did you receive treatment for your eating disorder?
    - What does this timeline look like?
3. When you sought eating disorder treatment, what type of health professional did you see and how often?
4. How was your [vegetarian, vegan] status perceived by the health professional?
    - Did you feel that your health professional was knowledgeable about eating disorders and [vegetarianism, veganism]?
5. Did your own perceptions of your [vegetarian, vegan] status change over treatment?
6. What are your thoughts on the quality of care of your treatment?
    - Do you think your quality of care was different from that of someone adhering to other ways of eating [omnivore, meat-eating]?
7. How might a health professional evaluate whether a client is a genuine [vegetarian, vegan] when seeking eating disorder treatment?

Note: Follow up questions were asked on the basis of participant answers. The interview guide questions provided the major topic areas to be explored.
