# Supplementary figures and images for: The Treatment Experiences of Vegetarians and Vegans with an Eating Disorder: A Qualitative Study
Source: Nutrients. 2025 Jan 18;17(2):345. doi: 10.3390/nu17020345 (PMC11768365; doi:10.3390/nu17020345)

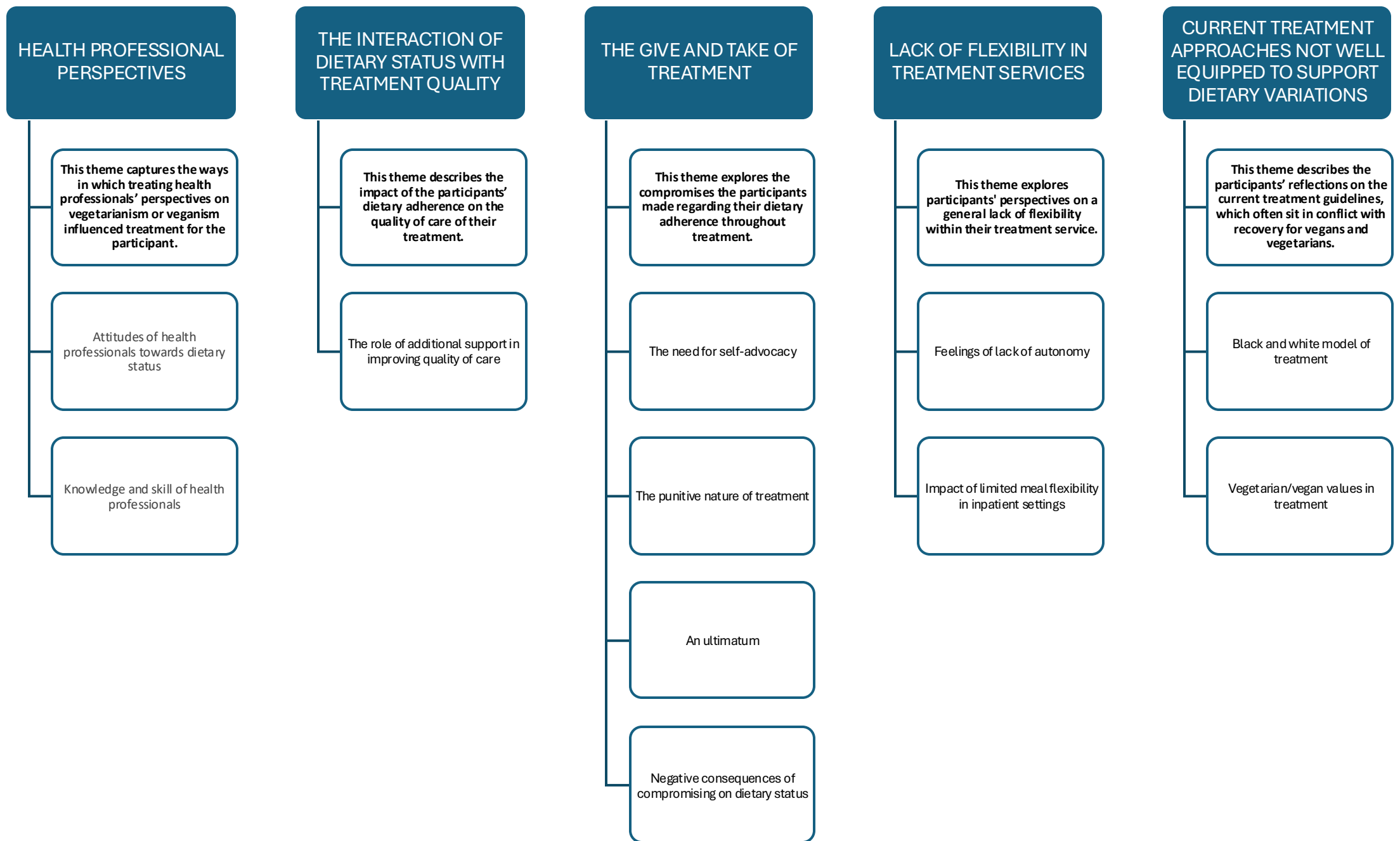

**Figure S1. Identified themes, subthemes, and their definitions**

Supplement: Supplementary file 1 [file nutrients-17-00345-s001.zip › nutrients-3403981-supplementary/Supplementary Materials - Figure S1.pdf]
